# Simulation-based mastery improves nursing skills in BSc nursing students: a quasi-experimental study

**DOI:** 10.1186/s12912-020-00532-9

**Published:** 2021-01-06

**Authors:** Roghayeh Mehdipour –Rabori, Behnaz Bagherian, Monirsadat Nematollahi

**Affiliations:** 1 Nursing Research Center, Kerman University of Medical Sciences, Kerman, Iran; 2 Department of medical- surgical nursing, Razi faculty of Nursing and Midwifery, Kerman University of Medical Sciences, Kerman, Iran; 3 Department of pediatrics and neonatal intensive nursing, Razi Faculty of Nursing and Midwifery, Kerman University of Medical Sciences, Kerman, Iran

**Keywords:** Clinical education, Nursing students, Simulation-based mastery model, Clinical skills

## Abstract

**Background:**

Clinical education is an essential part of nursing education. Selected clinical teaching methods influence the quality of education. Simulation-based mastery learning has been used to improve clinical skills among nursing students and may provide a novel way to enhance nursing skills. This study aimed to assess the effect of simulation-based mastery learning on the clinical skills of undergraduate nursing students from 2017 to 2019.

**Methods:**

This quasi-experimental study was conducted with two groups (the control and intervention). A hundred and five students were selected by random convenience sampling, and written consent was obtained. The intervention group participated in a simulation-based mastery learning intervention, and the control group received no intervention except for traditional training. The students of both groups completed the demographic information questionnaire and the checklist before and after the intervention. The results were analyzed by SPSS version 21 and descriptive and inferential statistics.

**Results:**

The results showed no significant differences between the two groups before the intervention (*p*> 0.05). In addition, students’ performance in the intervention and control groups improved significantly at the post-test compared with the baseline (*p*< 0.05). Furthermore, the Cohen test implied that the simulation-based mastery model used by the intervention group was significantly more effective than the traditional training used by the control.

**Conclusion:**

These findings showed that mastery learning was more effective in improving clinical skills in undergraduate nursing students. The results suggest that other nursing and health programs can be developed by implementing a mastery-based learning model.

## Introduction

Clinical education is an essential part of nursing and midwifery education [1]. Nursing educators try to create professional learning behaviors in nursing students and respond appropriately to specific clinical situations [2]. There is a trend in nursing education to adopt competency-based education (CBE) models. Anima and McCoy define competency as acquiring integrated knowledge, skills, values, and attitudes required for a competent nurse [3]. In addition, the development of nursing students with professional competence is one of the aims of nursing education [4]. Mastery is one of the competency-based models.

Mastery models differ from traditional curricula in defining progression as achieving a series of competencies [5]. Educators make a valuable contribution to the learning process by creating competency-based models that can enhance training [6]. In conclusion, students acquire the ability to do clinical skills [7].

Mastery is a new applied method for training students in the medical sciences, and it is one of the individual learning styles [8]. It originates from Carroll’s belief that if sufficient time is given to the full extent of education, the right education level will be achieved [9]. Mastery learning features frequent formative assessments to provide feedback and evaluate whether students have mastered an instructional standard [5]. However, some studies indicated that it was time-consuming [10] and caused student anxiety because of frequent evaluations [11]. Furthermore, Salvin showed that mastery learning had virtually no effect on student achievement. Still, it was necessary to be assessed by scholars and practitioners well-equipped in mastery learning until questionable aspects of this method were elucidated [12].

Şenel Elaldı found that the mastery learning model enhanced students’ understanding of what they have learned, and it was an opportunity for students’ development of learning [13]. Mastery models engage learners in contemplative practice by increasing the difficulty of repetitive tasks while providing coaching to guide their progress [14]. Furthermore, this method makes students more involved in the teaching-learning process [15]. For students failing to attain mastery, the assessment provides a diagnostic tool to develop an individualized learning plan to guide corrective action and to address deficiencies. Students who initially fail to demonstrate this skill have more opportunities (According to the curriculum) to take and pass the course [16]. Using a mastery model offers the potential for greater accountability, flexibility, and focus on learning process [17].

The advantages of this method are that students perform clinical skills correctly and fulfill professional responsibilities in the future [18]. Wayne et al. showed that mastery learning influenced CPR skills [19].

Frogameni et al. also indicated that symbolic mastery learning was an effective strategy in training residents to manage mechanical ventilators. Relying on traditional teaching methods in ICU may leave residents ill-equipped to handle patients receiving mechanical ventilation [20] safely. Cohen showed that the mastery learning method led to the acquisition of nasogastric tube skills in nursing students [21]. Based on the findings of a study, the mastery learning method is useful in nursing education [22]. In addition, Simulation-Based Mastery Learning improved central line maintenance skills of ICU nurses [23]. However, Roh showed that the mastery learning method did not significantly affect knowledge, self-efficacy scores, and the number of errors related to cardiopulmonary resuscitation skills in nursing students [24].

However, nursing students must combine knowledge from sociobiological and nursing sciences to make clinical decisions and manage different situations in the clinical settings [25]. Moreover, as the largest group of caregivers who deal directly with the patients, nurses can provide high-quality care through mastery of nursing skills [24].

However, there is little in the nursing literature about mastery model-based programs. Most Iranian nursing educators apply traditional learning-teaching methods, which are subject-centered, time-based with summative evaluation, and little feedback. They do not use the mastery method to train skills. Furthermore, Iranian educators do not have much information about mastery models. Regarding the benefits of the method mentioned above, the research team decided to assess the effects of simulation-based mastery on the clinical skills of B.Sc. nursing students.

## Methods

### Research design and setting

This quasi-experimental study was conducted with a pretest-posttest two-group design in the nursing department of Kerman University of Medical Science in Iran from 2017 to 2019. The Kerman University of Medical Science is the most prominent in the Southeast of Iran. The university provides education for undergraduate, postgraduate, and Ph.D. nursing students.

### Sampling method

Students were selected using convenience sampling and were then randomly divided into control and intervention groups by numerical table.

The inclusion criteria included the seventh- and eighth-semester nursing students who were not educated with the simulation-based mastery method previously. In addition, students had to pass the theoretical and practical courses such as courses in medicine, surgery, pediatric nursing, community health nursing, intensive care, and psychology.

In this study, the research population consisted of 115 eligible BSc students who met the inclusion criteria. Ten participants were excluded because of absence from training sessions.

In the nursing department of KMU, all nursing students had to pass prerequisite courses before taking the internship course, including theoretical and practical courses. The theoretical course includes the workshop on patient communication, nosocomial infections, nursing ethics, and a practical course includes common nursing skills trained in proficiency workshop, where advanced special moulage and other equipment are available for nurse students to practice the special nursing skills.

Students will take the practical course after completing the workshops. In addition, taking the internship course depends on passing (80% of the checklist) the clinical exam. All students of the nursing department had to take the same curriculum.

The research team selected nursing skills commonly used in nursing and agreed on the following four practical skills: suction, nasogastric tube feeding, packed cell transfusion, change of fluid box.

First, the study goals were explained to the participants. The students participated in the study with full consent and agreement. They were explained that attending or not participating in the study would not affect their educational process. The instructors in the two groups completed the demographic characteristic questionnaire and checklist skills before the intervention, and common nursing skills were assessed in two groups by a checklist.

### Intervention group procedure

The intervention began on the second day of the course. The intervention group members experienced a simulation-based mastery intervention in four common clinical skills (suction, nasogastric tube feeding, packed cell transfusion, change of fluid box).

First, the instructor performed each skill on the advanced moulage in the proficiency workshop. Then, the students practiced these skills and were assessed by the instructor, who could identify whether they learned the skill or not (diagnostic feedback) and what they needed to learn better (prescriptive feedback).

A list of student’s mistakes was provided for the relevant instructor and student. The instructors set specific goals for each student based on the deficiencies identified in the first stage. In this program, the instructor used supervisory and observational methods. The instructor observed students and completed checklists every day. The instructor re-evaluated the students through a checklist and re-identified some deficiencies listed in the checklist daily for 12 days (2 days a week). In addition, students, who initially fail to demonstrate the skill, have three more opportunities to pass the course. At the end of the course, the clinical skill scores were checked.

To determine the observer’s accurate judgment on the examination based on the checklist, two observers assessed the inter-rater reliability of the assessors’ scoring for each of the skills. A single-blind method was used so that the students involved in this study were not informed of the type of teaching methods and how they were placed in each group. In this study, the intervention group experienced a simulated mastery learning method.

### Control group procedure

Common nursing skills were trained to the control group students in a proficiency workshop during two sessions a week for 6 weeks.

The routine teaching method was as follows: the students were divided into groups of three individuals, and clinical skills were performed on the advanced moulage under the instructor’s supervision. In case of any question or mistake, the instructor, a facilitator, would address it. In this method, the instructor taught students, according to time-based and summative evaluation with little feedback.

The demographic information questionnaire and the checklist were used in this study to collect the data.

### Instruments

The demographic questionnaire contained information about age, sex, scale median, passed credits, and grade point average last semester.

The researchers used four nursing skill checklists for both groups (control and intervention). The checklists were extracted from a nursing book: Skill checklists for Tylor’s clinical nursing skills [26]. The suction checklist, the nasogastric tube feeding checklist, the packed cell transfusion checklist, and the change of fluid box checklist contain 19, 19, 13, and 21 items, respectively. Each item on the checklists is rated using three scales: unsatisfactory (score: 0), satisfactory (score: 1), and excellent (score: 2). The suction, nasogastric tube feeding, packed cell transfusion, change of fluid box checklists were scored 0–38, 0–38, 0–26, and 0–42, respectively. The total score ranges from 0 to 144.

The content validity of the checklists was confirmed by the broad consensus, and their reliability was 0.82 by using a pilot study and the Cronbach’s alpha coefficient showing good reliability.

In addition, medical-surgical nurses, pediatric nurses, and intensive care nurses in Kerman have attempted for 5 months to prepare and select nursing skill checklists.

### Data analysis

The collected data were analyzed using descriptive (frequency, percentage, mean and standard deviation) and inferential statistics. According to the Kolmogorov–Smirnov test results, the data of this study had a normal distribution. Thus, parametric tests were used. Furthermore, independent t-test was employed to compare the mean scores of skills between the intervention and control groups before and after the intervention. The paired samples t-test was also used to compare the mean scores of skills in each group before and after the intervention. *P*-values were considered statistically significant.

## Results

The participants in this study were 105 BSc nursing students of Kerman University of medical science. The participants were divided into two groups of intervention (*N*=53) and control (*N*=52). Students’ mean ages in the intervention, and the control groups were 23.88±2.06 and 23.38**±**1.78, respectively.

Most of the participants were female (38 individuals in the intervention group and 39 individuals in the control group). A majority of the participants were native (43 individuals in the intervention group and 42 individuals in the control group); most of them had no history of diseases and took good grade point averages in the last semester. No significant difference was found between the control and intervention groups in their demographic data (Table 1).

**Table 1:** Demographic characteristics of nursing students in intervention and control groups

Demographic characteristics	Mastery learning intervention	Common learning group	***p-***value
**age**	**μ ±SD** **23.88±2.06**	**μ ±SD** **23.38±1.78**	*P*> 0.05*
**Mean semester**	**16.4±1.55**	**16.01±2.03**	***P>*** **0.0**
**Passed unites**	**93.25±0.84**	**93.16±0.57**	
**Sex**			
**Female**	***N*** **=38 (71.96%)**	***N*=39 (73.07%)**	***P>*** **0.05**
**male**	***N*=15 (28.04%)**	***N*=13 (26.93%)**	
**Native**	***N*=43 (81.13%)**	***N*=42(80.7%)**	***P>*** **0.05**
**Nonnative**	***N*=10 (18.86%)**	***N=*10(19.3%)**	

*Qui square

The total mean scores of the participants’ clinical skills in the intervention group were 101.6±3.69 and 141.6±3.13 before and after the intervention, respectively. In addition, the total mean scores of clinical skills in the control group were 88.17±6.11 and 109.36 ± 4.71, respectively. Independent samples t-test showed a statistically significant difference between the two groups after the intervention (*P* < 0.05). The Cohen test also showed a statistically significant difference between them after the intervention (d=5.6).

In addition, the results of this study showed that the mean score of each of the clinical skills was not statistically significant between the control and the intervention groups before the intervention. However, a statistically significant difference was found between them after the intervention (Table 2).

**Table 2:** Comparison of the Mean scales inter and between the two groups

Clinical skills		Simulation mastery learning group	Common learning group	Statistic t* and p	cohen’s d
Suction (0–38)	Before after	26.46±1.51 37.20±0.95	24.9±1.11 27.85±1.30	0.1 t=−2.24 0.00 t =24.52	−0.43 4.78
statistic t** and *p*		0.000* t=−-46.13	0.00 t=−24.01		
NG tube feeding (0–38)	Before after	23.46±1.79 37.41± 0.49	23.85±1.30 28.075±1.5	0.35 t =−4.5 0.02 t =23.05	−0.87 4.58
statistic t** and *p*		0.00 t=−63.43	0.00 t=−14.56		
Packed cell transfusion (0–26)	Before after	17±1.33 25.52±1.05	17.79±1.26 20.79±0.71	0.24 T=-3.23 0.03 T=20.09	−0.62 3.91
Statistic t** and *p*		0.000* t=−44.43	0.00* t=−14.77		
Changing fluid box (0–42)	Before After	25.68±0.4 41.47±0.64	24.63±1.23 33.57±1.46	0.27 T=-3.2 0.001 T=22.13	−0.62 4.31
Statistic t** and *p*		0.02* t=−24.2	0.00* T=-22.18		
Total score (0–142)	Before After	101.6±3.69 141.6±3.13	88.17±6.11 109.36±4.71	0.13 T= −4.6 0.00 T=28.9	−.89 5.63
Statistic t** and *p*		0.02* t=−26.2	0.00* T=-27.18		

*independent t-test

**paired t-test

The mean suction scores of the intervention group participants were 26.46±1.51 and 37.20±0.95 before and after the intervention, respectively. The mean scores of suction in the control group were 24.9 ±1.11and 27.85±1.30, respectively. Independent samples t-test showed a statistically significant difference between the two groups after the intervention (*P* < 0.05).

By comparison, the mean scores of ng tube feeding in the intervention group were statistically significant before (23.46±1.79) and after the intervention (37.41± 0.49) (*p* < 0.05).

Furthermore, the results showed that the mean nasogastric tube feeding scores in the control group were 23.85±1.30 and 28.075±1.5 before and after the intervention, respectively. Independent samples t-test indicated a statistically significant difference between the two groups after the intervention (*P* < 0.05).

The mean Pack cell Transfusion scores of the intervention group participants were 17±1.33 and 25.52±1.05 before and after the intervention, respectively. The mean scores of Pack cell transfusion in the control group were 17.79±1.26 and 20.79±0.71, respectively. Independent samples t-test showed a statistically significant difference between the two groups after the intervention (*P* < 0.05).

The mean changing fluid box scores of the intervention group participants were 25.68±0.4 and 41.47±0.64 before and after the intervention, respectively. The mean scores of changing fluid box scores in the control group were 24.63±1.23 and 33.57±1.46, respectively. Independent samples t-test showed a statistically significant difference between the two groups after the intervention (*P* < 0.05).

## Discussion

This study was one of the few studies in Iran conducted on the effect of symbolic mastery learning on the clinical skills in undergraduate nursing students.

The results of this study indicated that the mean scores of skills in the control group were statistically significant before (22.04±1.22) and after the training program (27.29±1.17) (*P* < 0.05). In addition, the mean scores of skills in the intervention group were statistically significant before (25.4±1.27) and after the intervention (35.4±0.46) (*p* < 0.05). This study showed that mastery learning was more effective in achieving clinical skills than the traditional method (d=5.6).

Barusk indicated that mastery learning increased the nursing student’s knowledge and skills scores for physical examination. Furthermore, he reported that mastery learning promoted the general competency of the students [27].

Tang showed that mastery-learning intervention increased nurses’ clinical competencies [28]. Moreover, Schroedl reported that the mastery learning method was useful to identify the professional competence of nursing practice [14]. Extensive research evidence shows that mastery learning can have positive effects on student achievement. In addition, Amiruddin (2015) pointed to the positive effects of mastery learning that can help students increase their efforts and ultimately perform academic tasks better [29].

This result was in line with the results of the present study. Contrary to other teaching methods, this method helps the instructor know the deficiencies of the students, and the students know that they have enough time to learn the skills [30]. Educators tried to teach the students the knowledge and skills required for competent nurses. Moreover, in this teaching method, instructors can determine students’ learning needs [31]. According to this study, the instructor identified students’ learning problems in each intervention stage and retested them. In addition, students who initially fail to demonstrate the skill have three more opportunities to take and pass the course.

Repeated assessments of students at given intervals improved the quality of education, and the students were active in the learning process [27]. In this study, acquiring competency was based on a skills scale.

The instructors in this study also considered this approach time-consuming. They believed that this approach was challenging with many nursing students and the limitations of the laboratory facilities. Roberts et al. indicated that this approach was time-consuming due to the organization of various tests and the high volume of nursing education contents [32].

Mohd Hasril concluded that mastery learning strategies were significantly associated with increased learning in vocational training compared with traditional mastery models. Trainees mentioned that those who received faster feedbacks were more successful [29]. In this study, the trainees received their feedback immediately after each skill. The nursing students who received feedback could identify their deficiencies. Applying mastery learning methods is useful in clinical settings to empower nursing students, and mastery learning is considered a new paradigm in medical education [9]. In addition, the students acquired high-quality skills because of giving feedback along the teaching process. According to the experiences of instructors, some students have anxiety when they receive feedback. Thus, scientific and psychological support to the students improves their clinical skills. Evaluating this model and examining its strengths and weaknesses predisposed different students to apply it in various educational settings.

### Limitation

This study was done only in the nursing department of Kerman University of Medical Science so that the generalizability of the study data was limited to some extent. Because the study method was time-consuming, the effect of the mastery learning method on the practical course was studied. It is suggested that the mastery learning method be evaluated on both theoretical and practical courses.

The results indicated that the mastery learning model had more beneficial effects than the traditional method. Furthermore, our study showed that this model offered rich and in-depth learning opportunities for students. These results, therefore, can encourage nursing authorities to continue their training and development in the research methodology.

In addition, this study would be fruitful for future research to examine the effect of mastery learning on self-esteem, satisfaction, and competency of students.

## Conclusion

The study results showed that the implementation of the mastery learning method was more effective in training clinical skills in BSc students. In addition, the findings indicated that students in the mastery learning model group achieved higher grades in clinical skills than those who used the traditional method. In addition, the quality of learning improved in undergraduate nursing students in the simulation-based mastery learning group. Furthermore, it is a flexible and successful approach and enhances students’ skills.

## Data Availability

The datasets used and/or analyzed during the current study are available from the corresponding author on reasonable request.
